# Multimodal intervention program to improve hand hygiene compliance: effectiveness and challenges

**DOI:** 10.1186/s42506-020-00039-w

**Published:** 2020-03-23

**Authors:** Sihem Ben Fredj, Asma Ben Cheikh, Sana Bhiri, Hela Ghali, Salwa Khefacha, Lamine Dhidah, Latifa Merzougui, Mohamed Ben Rejeb, Houyem Said Latiri

**Affiliations:** 1 Department of Prevention and Care Safety, University Hospital Sahloul, 4011 Sousse, Tunisia; 2 Department of Epidemiology, University Hospital Ibn El Jazzar, 3100 Kairouan, Tunisia; 3 Faculty of Medicine of Sousse, University of Sousse, 4000 Sousse, Tunisia

**Keywords:** Hand hygiene, Compliance, Intervention

## Abstract

**Background:**

Hand hygiene (HH) is considered the most important measure to tackle the transmission of healthcare-associated pathogens. However, compliance with recommendations is usually low and effective improvement strategies are needed. We aimed to assess the effectiveness of an intervention targeting hand hygiene promotion among healthcare workers (HCWs).

**Methods:**

We conducted a pre-post interventional study design in the university hospital Sahloul, Sousse, Tunisia, from January 2015 to December 2016. The intervention program consisted of training sessions and distribution of posters of hand hygiene guidelines. To assess the evolution of HH observance at pre- and post-intervention, the same observation form was distributed and collected at healthcare workers’ workplace.

**Results:**

Of the 1201 and 1057 opportunities for hand hygiene observed among all categories of HCWs, overall compliance enhanced significantly from 32.1 to 39.4% (*p* < 0.001) respectively at pre- and post-intervention. Nurses were the most compliant with a significant improvement from 34.1 to 45.7% (*p* < 0.001) respectively at pre- and post-intervention. Furthermore, analysis by department showed significant improvement of compliance in orthopedic department (*p* < 0.001), maxillofacial-surgery department (*p* < 0.001), pediatrics department (*p* = 0.013), and emergencies (*p* = 0.038).

**Conclusion:**

This study showed the feasibility and effectiveness of a health-setting-based intervention to enhance hand hygiene observance in the context of a developing country.

## Background

In Tunisia, several studies have uncovered the alarming fact that up to 17% of inpatients develop healthcare-associated infections (HAIs) [1–3]. Patients sustaining a HAI compared with those who do not, have significantly higher morbidity, mortality, and length of stay [4–6]. That vicious circle of uncontrolled speeding up of HAIs and increasing financial losses would ultimately weaken healthcare systems especially in low- and middle-income countries, where little data are available. In developed countries, these infections costs an additional €7 billion per year in Europe, considering direct costs only [7] and USA $ 6.5 billion annually for the care of inpatients in the USA [8]. This alarming global burden is avoidable. It is well established that the hands of healthcare workers (HCWs) are the main way of pathogen transmission from one patient to another and within the healthcare environment during the healthcare delivery [9, 10]. Therefore, the key element in interruption of the HAIs spread is sustainable hand hygiene (HH). Evidence-based models and prospective studies backed the importance of HH adherence to decrease the HAIs occurrence and to improve the patient outcome [11]. However, HCWs usually comply poorly with recommendations particularly in settings with limited resources reflecting a gap between evidence and real practice [12]. Amazian et al. demonstrated that compliance with hand hygiene varied greatly between countries and settings but was globally low (27%) in 22 hospitals in four Mediterranean countries. The HH compliance rates were 52.8%, 32.3%, 18.6%, and 16.9%, respectively for Egypt, Tunisia, Algeria, and Morocco [13].

Promotion of effective measures to enhance HH adherence is a core component of the WHO initiative “save lives clean your hands” launched in 2009 in order to improve patient safety [14]. Thereby, WHO developed a multimodal implementation strategy and measures for hand hygiene [15] which proved its effectiveness and adaptability to different healthcare settings with different cultures, local specificities, and habits [16–23]. Our study aimed to demonstrate the feasibility and effectiveness of a health-setting intervention targeting hand hygiene promotion based on the WHO multimodal strategy in all the wards of University Hospital Center Sahloul in Sousse, Tunisia.

## Methods

### Setting

The study was conducted at the University Hospital Center (UHC) of Sahloul in eastern Sousse, Tunisia. It is a 690-bed tertiary-level teaching hospital with ten medical departments, ten surgical departments, and three laboratories. It is supported by 1141 healthcare professionals; among them are 173 physicians and 647 paramedical staff.

### Sample for study

Target population was all HCW categories (physicians, nurses, and housekeeping staff) agreeing to participate. All hospital departments were included except for operating theaters, laboratories, and administrative units. The included departments were the Medical Intensive Care Unit, Surgical Intensive Care Unit, departments of General Surgery, Internal Medicine, Cardiology, Orthopedics, Physical Medicine, Nephrology, Gastrology, Cardio-vascular and Thoracic Surgery, Maxillofacial Surgery, Dental Medicine, Neurology, Emergencies, Neurosurgery, Urology, and Pediatrics department.

According to the WHO recommendations for sample size determination [24], the minimal sample size required was 992 observations. The required number of opportunities to be observed was at both time periods, and based on an improvement in the hand hygiene compliance of 10% between the pre and post intervention. To compensate for possible nonresponse, a total of 1190 observations were planned for the study.

### Study design

**Fig. 1:**
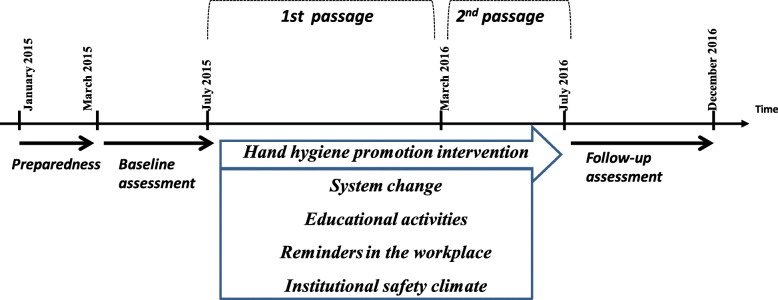
The study design of the interventional study at the University Hospital Sahloul, Sousse, Tunisia (2015-2016)

This study adopted a pre-post interventional study design with one group of HCWs working in all the departments of UHC Sahloul, Sousse, from January 2015 to December 2016. It was designed and reported according to the WHO hand hygiene improvement strategy [15], which has been implemented and succeeded in many institutions across the world [23]. We adapted the strategy as per our institution organizational characteristics (Fig. 1). The global approach was based on the following 4 steps [25].

#### Step 1, preparedness

Two months prior to the first evaluation of HH compliance, the Department of Prevention and Care Safety ensured the institution preparedness by providing the necessary resources, making available alcohol-based hand rub at the point of care, reviewing the main issues, and clarifying the plan schema of the strategy. For that, we informed all departments’ heads and the general director of the hospital about the study.

#### Step 2, baseline assessment

The research team conducted a pre-intervention baseline assessment of HH compliance during 2 months. The trained data collectors observed directly the selected professionals, for 2 h daily during the morning shift. Each observation was broken down into sessions of 20 min distributed equally throughout the study duration. HCWs were not informed about neither the actual goal of the observations nor the schedule of the observations’ time. They were not aware of when exactly these observations were being made.

#### Step 3, intervention

The intervention program was launched in step 3.

The intervention program started in January 2016 and lasted 7 months. It comprised two periods of passages in order to reinforce the educational program for hand hygiene promotion. The intervention strategy was based on the key components of the WHO multimodal strategy [25]:

##### System change

We used a checklist to review once a month the availability of alcohol-based hand rub, the liquid soap, washbasins, washbasins/bed ratio, and hand towels for single use. Alcohol-based hand rub, liquid soap, and towels were distributed at points of patient care not available. The damaged where they were washbasins were either fixed or replaced.

##### Educational activities

The implementation approach of the training program was based on the WHO tools for HH promotion [26]. Whereby, we rolled out open sensitization days, educational sessions, showing educational films followed by interactive discussion and presentation of the pre-intervention results. We delivered an attendance certificate for encouraging HCWs to participate in the workshops. These different methods targeted an awareness-raising about the burden of the hospital acquired infections (e.g., morbidity, mortality, and costs), the concept of HH and its key role in the prevention of hospital acquired infections and the correct techniques of HH.

##### Reminders in the workplace

HH leaflets were distributed to each department and posters were bonded in strategic areas of the hospital departments. They included key messages and emphasized the HH importance as the cornerstone of infection control. They also showed the techniques of hand washing with soap and water or cleaning with alcohol-based hand rub.

##### Institutional safety climate

We introduced a compelling communication to motivate the stakeholders to be involved in creating an environment that promotes and encourages patient safety. We were seeking to get the support of all HCWs. Therefore, we tried to obtain a formal and clear commitment from the senior hospital managers for the promotion of HH in order to maximize the HCWs involvement in this project.

#### Step 4, post-intervention assessment

This step consisted of the follow-up and feedback period. The post-intervention evaluation of the HH compliance was continued.

### Definition of terms

Five indications/moments for HH are based on those defined by the WHO guidelines [27].

A moment or indication is when there is a perceived or actual risk of pathogen transmission from one surface to another via the HCWs hands (gloved or ungloved) while undertaking a succession of tasks [28].

According to the WHO guidelines, the hands should be washed with soap or rubbed with alcoholic disinfectant [29]:

- Moment 1: before patient contact
- Moment 2: before a procedure or an aseptic task
- Moment 3: after a body fluid exposure risk
- Moment 4: after touching a patient
- Moment 5: after touching patient surroundings

Opportunity for HH is a situation whenever one of the moments for hand hygiene is present and observed during patient care. Two indications for HH may co-occur for one opportunity.

Compliance with HH was defined as either washing the hands (gloved or ungloved) with water and plain soap or rubbing the hands with an antiseptic solution when an opportunity occurred. Departure from the room after patient care without HH and failure to remove gloves after patient contact or contact between a dirty and a clean body site on the same patient were considered noncompliance [30]. HH compliance, the main outcome measure, was calculated as the proportion of HH indications for which HCW performed a correct action [31]:

$$ \mathrm{HH}\ \mathrm{compliance}=\frac{\mathrm{number}\ \mathrm{of}\ \mathrm{acts}\ \mathrm{of}\ \mathrm{HH}\ \mathrm{when}\ \mathrm{the}\ \mathrm{indication}\ \mathrm{exists}\times 100}{\mathrm{total}\ \mathrm{number}\ \mathrm{of}\ \mathrm{HH}\ \mathrm{opportunities}} $$

### Instruments of measurement

We assessed HCWs adherence to HH guidelines at pre and 6-months post-intervention with a validated tool. It was constructed by the WHO [31], and composed of five parts. The first part was about the general information of the study setting and sessions’ execution. The second part included the professional’s category, the indication, and the HH action whether it was a hand washing, alcohol-based hand rub, or no action was taken. Wearing gloves was considered to be no action.

### Data analysis

Statistical analysis was carried out using the program SPSS v. 21 software for windows. The absolute and relative frequencies were given for the qualitative variables. Proportions were compared by using chi-square tests to compare the HH compliance among HCWs according to their categories, specialties, and departments. Multiple logistic regression analysis was used in order to seek potential determinants of HH compliance. The adjusted odds ratios (aOR) and 95% confidence interval (CI95%) were calculated. The significance level was set at 0.05.

## Results

A total number of 2258 opportunities for HH were observed, 1201 at the baseline assessment and 1057 opportunities in the 2 months of observation that followed.

Overall compliance enhanced significantly from 32.1 at baseline to 39.4% (*p* < 0.001) at follow-up. We observed striking differences in the level of compliance among the three professional categories. Markedly improved adherence was recorded among nurses. Their compliance improved significantly from 34.1 to 45.7% (*p* < 0.001) respectively at pre- and post-intervention. HH compliance among doctors decreased insignificantly from 30.7 in 2015 to 23.1% in 2016 (*p* = 0.06). Housekeeping staff recorded the lowest HH compliance which dropped from 19.8 to 16.1% (Table 1).

**Table 1:** Hand hygiene compliance among health care professionals, before and after intervention, at University Hospital Sahloul, Sousse, Tunisia 2015–2016

	Pre-intervention		Post-intervention		***p*** value
Subgroups	Compliance ***n*** (%)	CI95%	Compliance ***n*** (%)	CI95%	
**Overall**	**385 (32.1)**	29.5 – 34.8	416 (39.4)	36.4–42.3	**< 10**^**−3**^
**Professional category**					
Nurses	269 (34.1)	30.8–37.5	355 (45.7)	42.2–49.3	**< 10**^**−3**^
Physicians	99 (30.4)	25.7–36.0	52 (23.1)	17.8–29.2	0.06
Housekeeping staff	17 (19.8)	12.3–29.1	9 (16.1)	8.1–27.4	0.57
**Medical speciality**					
Intensive Care	197 (41.4)	36.2–45.1	129 (48.9)	42.7–55.0	**0.029**
Surgery	63 (20.9)	16.8–26.3	158 (34.6)	30.2–39.1	**< 10**^**−3**^
Medicine	107 (29.3)	24.7–34.3	110 (36.2)	30.7–41.7	**0.05**
Emergency	18 (36.0)	23.2–50.8	19 (59.4)	40.7–75.7	**0.038**
**Department**					
Nephrology	22 (31.4)	21.1–43.7	5 (9.6)	3.5–21.7	**0.004**
Physical Medicine	15 (16.5)	9.8–26.0	10 (18.2)	9.5–31.3	0.79
Urology	10 (24.4)	12.9–40.6	22 (24.4)	16.2–34.8	0.99
Neurology	17 (37.0)	23.5–52.4	10 (31.3)	15.7–50.1	0.60
General Surgery	13 (24.1)	13.9–37.9	21 (33.3)	22.2–46.4	0.27
Cardiology	30 (35.3)	25.4–46.4	33 (35.1)	25.7–45.7	0.98
Orthopedics	4 (7.0)	2.2–17.8	48 (37.8)	29.4–46.8	**< 10**^**−3**^
Surgical Intensive Care	56 (35.7)	28.3–43.7	31 (37)	26.8–48.1	0.85
Gastrology	24 (29.6)	20.2–40.9	13 (40.6)	24.2–59.2	0.26
Neurosurgery	11 (26.8)	14.7–43.2	24 (45.3)	31.8–59.4	0.06
Maxillofacial Surgery	2 (4.3)	0.7–15.7	28 (46.7)	33.8–59.9	**< 10**^**−3**^
Internal Medicine	25 (36.8)	25.6–49.3	17 (51.5)	33.8–68.8	0.15
Thoracic and Cardiovascular Surgery	28 (41.2)	29.5–53.7	34 (53.1)	40.3–65.5	0.17
Medical Intensive Care	36 (38.7)	28.9–49.4	15 (50.0)	31.6–68.3	0.27
Pediatrics	58 (47.2)	38.1–56.3	78 (63.0)	53.7–71.2	**0.013**
Dental Medicine	16 (55.2)	35.9–72.3	8 (25.0)	12.1–43.7	**0.016**

Improvement was observed across all medical specialties. In medicine, HH compliance averaged 29.3% at pre-intervention and 36.2% at post-intervention. In surgery, HH compliance averaged 20.9% at pre-intervention and 34.6% at post-intervention. The intensive care units, whether they were medical or surgical, had the highest compliance rate in the two periods of the study (Table 1).

Furthermore, analysis by department indicated significant improvement of HH compliance from baseline to the intervention period within the majority of hospital departments. It increased significantly in the orthopedic department from 7 to 37.8% (*p* < 0.001), in the maxillofacial surgery department from 4.3 to 46.7% (*p* < 0.001), in the pediatric ward from 47.2 to 63% (*p* = 0.013), and in the emergency department from 36 to 59.4% (*p* = 0.016). Nevertheless, HH compliance declined significantly in nephrology department from 31.4 to 9.6% (*p* < 10^−3^).

HH compliance was enhanced across all indications for HH; however, it was only significant for “before aseptic task” (Table 2).

**Table 2:** Hand hygiene compliance according to the WHO 5 indications, before and after intervention, at University Hospital Sahloul, Sousse, Tunisia 2015–2016

	Pre-intervention			Post-intervention			
Indications for hand hygiene	Hand hygiene opportunities***n***	Compliance*** n*** (%)	CI95%	Hand Hygiene opportunities ***n***	Compliance*** n*** (%)	CI95%	***p*** value
Before patient contact	453	107 (23.6)	19.6–27.6	340	81 (23.8)	20.1–29.7	0.94
Before aseptic task	294	71 (24.1)	19.1–29.1	196	210 (38.1)	34.4–48.5	0.01
After body fluid exposure risk	73	34 (46.6)	34.9–58.5	40	20 (50.0)	36.8–70.7	0.72
After patient contact	407	206 (50.6)	44.6–54.5	438	213 (48.6)	44.9–54.5	0.56
After contact with patient surroundings	158	44 (27.8)	21.1–35.6	83	23 (27.7)	22.5–42.7	0.89

After adjusting all variables to each other in a logistic regression analysis model, the study showed that HCWs were more significantly (aOR = 1.34, CI95% 1.11–1.62) compliant after the HH intervention; nurses were significantly (aOR = 2.03, CI95% 1.66–2.49) more compliant compared with physicians and other HCWs; compliance was more significant in the indication before aseptic task (aOR = 1.56, CI95% 1.25–1.94) (Table 3).

**Table 3:** Binary logistic regression model of potential factors determining hand hygiene compliance at University Hospital Sahloul, Sousse, Tunisia 2015–2016

Variable	aOR	CI95%
**Event**		
Pre-intervention	Reference	
Post-intervention	1.34	1.11–1.62
**HCWs**		
Physicians and other HCWs	Reference	
Nurses	2.03	1.66–2.49
**Indications**		
Other indications	Reference	
Before aseptic task	1.56	1.25–1.94

***aOR*** adjusted odds ratio, *CI95%* 95% confidence interval

## Discussion

As far as we know, this study was the first to report the implementation of the WHO hand hygiene improvement strategy using a multifaceted approach in a Tunisian healthcare setting. At baseline, HH compliance (31.6%) of HCWs in the university hospital of Sahloul was comparable to that shown in the literature [18, 32–34]. However, it was still far from other results [20, 35, 36]. Remarkably, the intervention program resulted in a significant improvement in HH compliance which reached the 39.4% in post-assessment. However, the improvement was observed only among nurses, and a slight decrease in HH compliance was recorded among physicians. Similarly to most studies, the nurses showed a higher HH compliance than did the other professionals [19, 37–39]. In the present study, the poor HH adherence among physicians comparing to nurses may be explained by the limited attendance of doctors in training sessions. Hence, making changes to HH adherence is challenging in our context, particularly to face defective behaviors and routines that may be already established with HCWs. Although the HH is a simple act to do and a core component in infection control, it seems that it is hardly incorporated into clinical practice especially that physicians showed no significant change in HH compliance [40]. This phenomenon would be the consequence of potential interferences that impede the best HH practice [41]. The behavioral determinants were conceptualized as two themes by Maura et al. in a systematic qualitative literature review, according to a theoretical background. The first component was the motivational factors including the social influences, acuity of patient care, self-protection, and use of cues. The second component was the perception of the work environment whether it concerns the resources, the knowledge, the information, or the organizational culture [42]. The contributing factors could be also classified according to their type: individual or organizational determinants. The individual determinants’ concern mainly the perception of HAIs risk [43], the knowledge and skill gap [40] or else the forgetfulness [44], dermatology problems, and poor acceptance [45] and obviously, HCWs compliance is influenced by senior’s role model [46]. Moreover, hand hygiene adherence among HCWs is frequently suboptimal and resistant to improvement as shown by Larson et al. [47]. The organizational determinants could be summarized by the work environment characteristics such as the information accessibility and access to HH resources, especially at patient bedside [42] and the high workload [30]. In addition, as a developing country, other factors should be considered in the implementation of infection control program including costs, procurement constraints, deficient infrastructure, cultural issues, and lack of knowledge [48]. Therefore, a multidimensional approach is the appropriate strategy to face such multiple constraints. However, the high workload was a heavy barrier in our health setting. First, the current personnel number is limited in relation to the growing demand for healthcare [49]. Second, the Tunisian government adopted a rigid strategy in recent years. Accordingly, we face challenges backfilling missing personnel. Third, we noted a mismanagement of the available human resources following the administration laxity in particular after the revolution in 2011. Nevertheless, the overall enhancement of the HH compliance was promising. Our intervention to positively influence HH behavior was effective as we hoped. The same trend was observed in most departments. Similar results were shown through literature around the world [1, 10, 12, 13, 15, 17, 29–33] reflecting the ability of multimodal interventions to raise awareness about the HH importance. However, it was not always the case. Some studies concluded that the intervention does not affect HH compliance [50, 51].

### Limitations of the study

Strengths of our study include the participation of all hospital departments, the use of the WHO intervention strategy that is highly reproducible and sufficient time study (beyond 1 year) to demonstrate significant changes. The main limitations of the study were the lack of control group since it was a single-center study and thus, the educational program would have spread easily. Besides, the Hawthorne effect may change the HCW behavior when they are aware of a professional observing them. This phenomenon has the potential to add bias to an outcome.

## Conclusion

Our study revealed the feasibility and effectiveness of a health-setting-based intervention to enhance hand hygiene observance in the context of a developing country. It suggests the need to incorporate the HH training as a part of the academic course and the professional diploma. A deeper analysis should be performed to further assess the determinant factors of compliance with hand hygiene. Future studies also should determine whether sustainable intervention program could slow the HAI transmission by targeting the HH compliance and obviously regular maintenance of the medical and paramedic equipment.

## Data Availability

The data used to support the findings of this study are available from the corresponding author upon request.
